# Sociodemographic characteristics associated with parenthood amongst patients with a psychotic diagnosis: a cross-sectional study using patient clinical records

**DOI:** 10.1007/s00127-022-02279-x

**Published:** 2022-04-21

**Authors:** Jessica Radley, Jane Barlow, Louise C. Johns

**Affiliations:** 1 Department of Psychiatry, University of Oxford, Warneford Hospital, Warneford Lane, Oxford, OX3 7JX UK; 2 Department of Social Policy and Intervention, University of Oxford, Barnett House, 32-37 Wellington Square, Oxford, OX1 2ER UK

**Keywords:** Psychosis, Parents, Cross-sectional, Clinical notes, Identification of dependants, Community mental health services

## Abstract

**Purpose:**

Estimates of parenthood in individuals with psychosis range from 27 to 63%. This number has likely increased due to the introduction of newer anti-psychotics and shorter hospital stays. The problems of psychosis can affect patients’ capacity to offer the consistent, responsive care required for healthy child development. The following research questions were assessed: (1) what proportion of these patients have their children correctly recorded in their clinical notes, (2) what proportion of patients in secondary care with a psychotic diagnosis have children, and (3) what sociodemographic characteristics are associated with parenthood in this population.

**Methods:**

This study used CRIS (Clinical Record Interactive Search) to search for patients with a diagnosis of non-affective or affective psychosis (F20–29, F31.2 or F31.5) within a UK NHS Trust. A binomial regression model was fitted to identify the variables associated with parenthood.

**Results:**

Fewer than half of the parents in the sample had their children recorded in the correct field in their clinical notes. Of 5173 patients with psychosis, 2006 (38.8%) were parents. Characteristics associated with parenthood included being female, older age, higher socioeconomic status, renting or owning, having ever been married, being unemployed, not being White (British) and not having a diagnosis of schizophrenia.

**Conclusion:**

Over one-third of patients with psychosis were parents, and the study indicates that not all NHS Trusts are recording dependants accurately. Many variables were strongly associated with parenthood and these findings may help target interventions for this population.

**Supplementary Information:**

The online version contains supplementary material available at 10.1007/s00127-022-02279-x.

## Introduction

The positive and negative symptoms of psychosis and side effects from antipsychotic medication can affect a parent’s capacity to look after their child [1]. Furthermore, the children of parents with psychosis are more likely than the children of parents without a mental health diagnosis to have behavioural and psychological difficulties [2] and are at an increased likelihood of taking on a caring role for their parents or siblings [3].

### Recording of children on patients’ clinical records

Parents with psychosis are often reluctant to seek help due to fear of being criticised as a parent or the possibility of social services involvement [4]. As a result of this, and of service providers being hesitant to ask about their children, dependants are often not present in their parents’ clinical records, making them invisible to services [5]. Policies in Norway [6], Sweden [7] and Australia [8] now require that adult mental health services record the presence of children accurately and work to meet the needs of the whole family. In the UK, the ‘Think Family’ initiative [9] called for improvement in the identification of patients’ children to signpost these families to relevant services and to safeguard the children as necessary.

### Global estimates of parental psychosis and factors associated with parenthood

An accurate estimate of how many people with psychosis are parents, and what characteristics are associated with parenthood, is needed to inform interventions for these families. The most recent estimates of parenting amongst people with psychosis have been conducted in Australia [10] and Germany [11], which were 27% and 38%, respectively. The most recent study in the UK was conducted over 20 years ago and reported that 63% of the 246 women with psychosis in a secondary care sample were mothers [12]. More recent estimates of the number of parents in UK adult mental health services have looked at any mental health diagnosis e.g. [13, 14], rather than psychosis specifically. The current rate is likely to be different since newer anti-psychotics and shorter hospital stays have increased fertility and opportunity to have children, respectively [15, 16].

It is also necessary to investigate factors that are associated with parenting status within those with a psychotic diagnosis. Certain characteristics have previously been shown to be associated with a better quality of care from parents with psychosis. For example, social class and lone parenthood [17] as well as illness severity [18]. By ascertaining which factors are associated with parenthood alongside the knowledge of which factors are associated with quality of care in parents, this will provide more information on the needs of parents with psychosis and inform more targeted interventions. Factors such as gender, age, marital status, and accommodation have previously been shown to be associated with parenting status [12, 19, 20]. This study will investigate these characteristics in a UK sample as well as others where there is mixed evidence for their association including diagnosis, ethnicity, and employment [12, 19, 20].

This study used CRIS, the Clinical Record Interactive Search, to estimate the proportion of patients with psychosis in an NHS Trust who have a child, to examine the recording of child details in UK electronic health records, and to understand some of the characteristics of these families.

### Research questions

1. What proportion of children are recorded in the correct structured field of their parents’ clinical notes?
2. What proportion of patients within an NHS Trust with a psychotic diagnosis have a child?
3. What are the sociodemographic factors that characterise being a parent with a psychotic diagnosis?

## Methods

This is a cross-sectional study using de-identified patient records from a UK mental health service case register.

### CRIS (clinical records interactive search)

CRIS is a database that contains over 2 million de-identified patient records from 14 NHS trusts across the UK, and allows the analysis of both structured and free-text data in an individual’s clinical records [21, 22]. This study accessed data from one of these Trusts.

### Study cohort

The CRIS system was searched on February 14th 2019 for all patients who had at any time been given an ICD 10 diagnosis of psychosis (F20–29, F31.2 or F31.5) or whose assigned cluster level indicated the presence of psychotic symptoms (cluster levels 10–14 and 16–17), yielding 5764 participants. To produce an estimate of parenthood at one point in time, those who had died on or before February 14th 2019 were removed from the sample, giving a final sample of 5173 participants.

### Study outcome

**Fig. 1:**
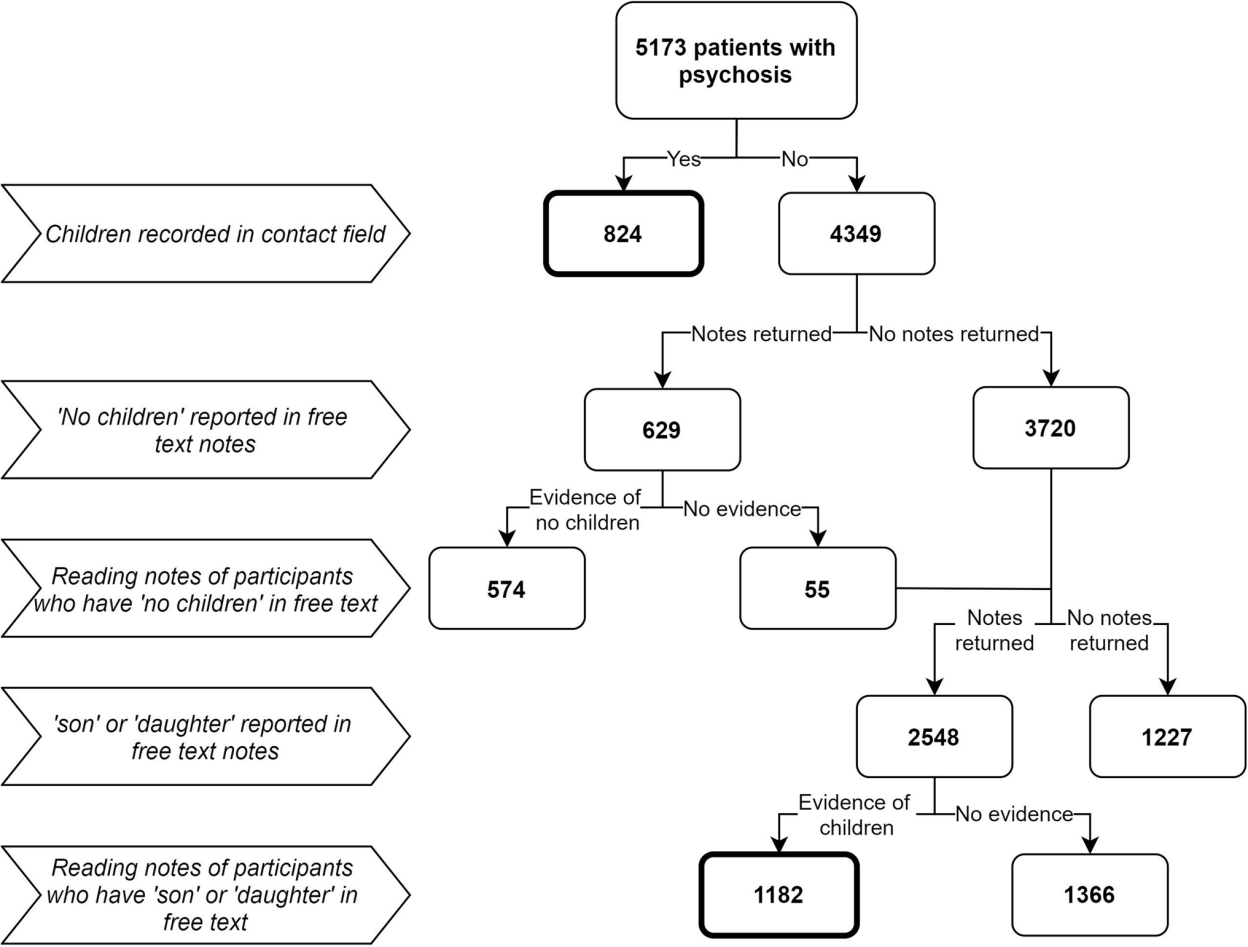
Identifying children in clinical notes

The primary outcome in this study was parent status (1 = participant is a parent; 0 = participant is not a parent). The sample was searched to see if any child details had been entered in the patient’s ‘contacts’ field, where the relationship was listed as ‘son’, ‘daughter’, ‘dependant’ or ‘stepchild’ (see Fig. 1). Children were defined as biological children or step-children of any age and it was not a requirement for them to be currently living with or cared for by the parent. The primary researcher then collected the free-text clinical notes of the patients who did not have children recorded in their ‘contacts’ field to determine whether any of these patients had children mentioned in their free-text clinical notes.

To gain an accurate estimate of how many patients in the sample had children, searches were then conducted in patients’ free-text clinical notes to identify any further children. A pilot search of ‘son’, ‘daughter’ and ‘child’ returned many irrelevant notes, and therefore, it was decided to search the notes for ‘no children’ first, to identify non-parents, and then search the remaining notes for ‘son’ or ‘daughter’.

For searches in the free-text clinical notes, the following process was followed (see also Fig. 1):

1. An initial search of free-text notes was conducted for the phrase ‘no children’ amongst the participants who did not have any children recorded in the ‘contacts’ field.
2. The notes returned from this search were read to confirm this did indeed mean that these participants did not have children or if ‘no children’ was referring to something other than the patient’s parenthood. These notes were also searched for the words ‘son’ or ‘daughter’ to check if the patient had not later become a parent before February 14th 2019.
3. A second search of free-text notes was conducted for any instances of the words ‘son’ or ‘daughter’ amongst those participants who did not have any children recorded in the ‘contacts’ field and did not have any notes returned from the initial search of free-text notes.
4. Each of these clinical notes was read to confirm the presence of children and extract data on the number, ages and genders of children, where it was reported.
5. The participants who did not have any clinical notes returned from these two searches were checked to see if their clinical notes were indeed populated, where the presence of a child might have been recorded. Once this was confirmed, these participants were assumed not to have any children.

### Independent variables

The following variables were extracted from the structured fields of all participants: gender, date of birth, ethnicity, marital status, employment, accommodation, LSOA (Lower Layer Super Output Area) marker, smoking status, ward stays and diagnosis.

Date of birth was used to derive participants’ ages on February 14th 2019. Ethnicity, marital status, employment, accommodation, smoking status and diagnosis were collapsed into broader categories to avoid small cell counts. LSOAs are geographic areas in the UK with an average population size of 1500, which can be linked to postcodes. Each LSOA marker was combined with the Office for National Statistics Index of Multiple Deprivation (IMD) [23] allowing each participant to be ranked against others living in different LSOAs in terms of deprivation. The IMD is based on 39 separate indicators across the seven domains of income, employment, education, health, crime, barriers to housing and services, and living environment, and is the official measure of relative deprivation in England [23]. These rankings were separated into nine equally sized groups of decreasing deprivation levels.

If multiple entries had been recorded for a participant on any variable, the most recent entry was selected to be used in the analysis.

### Analysis

To examine the sociodemographic differences between parents and non-parents, the relationship between parent status and each independent variable was tested individually using a *t*-test if the variable was continuous or ordinal, or a Chi-squared test if the variable was discrete or categorical. Those with missing data for the variable in question were first included and then excluded to determine whether it was the missingness that was significant between the two groups.

A binomial regression model was then fitted to determine which variables were most associated with whether a participant was a parent or not. There was a large amount of missing data for some variables, and due to nature of administrative data, it was assumed that this missingness was not at random (MNAR), whereby the missingness of a variable is related to the variable itself. Missing values for each variable were grouped into an additional category (labelled ‘unknown’) for the analysis. Although the missing indicator method is not usually recommended since it can introduce bias, it has been shown to be a good option when data are MNAR compared to methods such as multiple imputation or listwise deletion [24].

As a sensitivity analysis, the modelling was refit on a subset of the study cohort that excluded any participants without dependants (i.e. all children were 18 and over). This compared parents with dependant children and non-parents. All statistical analyses were performed in R (v4.0.2).

## Results

### Accuracy of recording of patients’ children in their clinical notes

Among the study cohort (*n* = 5173), 824 had children recorded in the structured field ‘contacts’ (Fig. 1). After searching the remaining 4349 for the phrase ‘no children’ in their free-text clinical notes, 629 records were returned and after reading these notes, 574 were confirmed not to have any children. The free-text search ‘son’ or ‘daughter’ returned 2548 participants’ clinical notes and, after reading these, a further 1182 showed evidence of having a child.

### Proportion of parents in the sample

In total, 2006 (38.8%) out of 5173 patients with psychosis were reported to have children. The remaining 3167 (61.2%) were assumed not to have children after extensive searching (Fig. 1).

### Demographic details of children

The 2006 parents in the sample had 3745 children in total. The mean average number of children was 1.87 per parent and the median value was 2. Most parent participants did not have any dependant children (see Supplementary table 1). Over two-thirds (67.2%) of parents with a least one dependant had their child’s details recorded correctly in the ‘contacts’ field of their notes, and just over a half (54.9%) of parents of non-dependants had them recorded in the ‘contacts’ field. The demographic details of the children are presented in Supplementary table 2. Some of these details were available from the CRIS data; however, the majority of them were obtained through reading patients’ clinical notes. Although it was often clear whether the child was under or over the age of 18, it was less likely that the exact age of the child would be acquired through reading the notes.

### Demographic details of participants

Table 1 shows the sociodemographic characteristics of parents and non-parents in the sample.

**Table 1:** Participant demographics

	Parents *n* (%) Total = 2006	Non-parents *n* (%) Total = 3167
**Gender**		
Male	764 (38.08%)	2077 (65.58%)
Female	1231 (61.37%)	1053 (33.25%)
Unknown	11 (0.55%)	37 (1.17%)
**Age**		
13 to 19	1 (0.05%)	122 (3.85%)
20 to 29	78 (3.89%)	739 (23.33%)
30 to 39	292 (14.56%)	728 (22.99%)
40 to 49	440 (21.93%)	627 (19.80%)
50 to 59	462 (23.03%)	493 (15.56%)
60 to 69	310 (15.45%)	245 (7.74%)
70 to 100	412 (20.54%)	176 (5.56%)
Unknown	11 (0.55%)	37 (1.17%)
**Ethnicity**		
White—British	1195 (59.57%)	1984 (62.65%)
Asian or Asian British	180 (8.97%)	223 (7.04%)
Black or Black British	103 (5.14%)	157 (4.96%)
Mixed race	48 (2.39%)	97 (3.06%)
White—other	151 (7.53%)	218 (6.88%)
Any other group	21 (1.05%)	57 (1.80%)
Unknown	308 (15.35%)	431 (13.61%)
**Marital status**		
Single	465 (23.18%)	1967 (62.11%)
Married/civil partner	572 (28.52%)	199 (6.28%)
Divorced/separated/widowed	394 (19.64%)	95 (3.00%)
Unknown	575 (28.66%)	906 (28.61%)
**Employment**		
Employed	129 (6.43%)	198 (6.25%)
Unemployed	99 (4.94%)	116 (3.66%)
Retired	305 (15.20%)	126 (3.98%)
Receiving benefits	169 (8.42%)	268 (8.46%)
Student	2 (0.10%)	93 (2.94%)
Unknown	1302 (64.91%)	2366 (74.71%)
**Accommodation**		
Owning	245 (12.21%)	132 (4.17%)
Renting	624 (31.11%)	784 (24.75%)
Supported living	149 (7.43%)	318 (10.04%)
Temporary or prison	147 (7.33%)	432 (13.64%)
Unknown	841 (41.92%)	1501 (47.40%)
**Smoking**		
Current smoker	448 (22.33%)	721 (22.77%)
Non-smoker	529 (26.37%)	727 (22.95%)
Ex-smoker	123 (6.13%)	171 (5.40%)
Unknown	906 (45.17%)	1548 (48.88%)
**Ward stays**		
None reported	892 (44.47%)	1565 (49.42%)
1	390 (19.44%)	576 (18.19%)
2 or more	724 (36.09%)	1026 (32.39%)
**ICD diagnosis**		
F20—schizophrenia	693 (34.55%)	1417 (44.74%)
F21—schizotypal disorder	11 (0.55%)	16 (0.51%)
F22—delusional disorder	159 (7.93%)	125 (3.95%)
F23—acute psychotic disorder	161 (8.02%)	243 (7.67%)
F25—schizoaffective disorder	273 (13.61%)	367 (11.59%)
F28—other nonorganic psychotic disorder	24 (1.20%)	46 (1.45%)
F29—psychosis not otherwise specified	118 (5.88%)	207 (6.54%)
F31.2 and F31.5—bipolar with psychotic symptoms	183 (9.12%)	161 (5.08%)
Psychosis indicated through cluster level	384 (19.14%)	585 (18.47%)

The two groups were significantly different in terms of age (*t*(4178.5) = − 29.6, *p* < 0.001), gender (*X*^*2*^(2, *N* = 5173) = 394.05, *p* < 0.001), ethnicity (*X*^*2*^(6, *N* = 5173) = 17.76, *p* = 0.007), marital status (*X*^*2*^(3, *N* = 5173) = 1162.9, *p* < 0.001), accommodation (*X*^*2*^(4, *N* = 5173) = 188.41, *p* < 0.001), employment (*X*^*2*^(5, *N* = 5173) = 261.06, *p* < 0.001), and diagnosis (*X*^*2*^(8, *N* = 5173) = 102.87, *p* < 0.001), both when including and excluding those with missing data. In terms of directionality, parents were older than non-parents, and a higher proportion of parents were female, married or divorced, retired or unemployed, owning or renting. A lower proportion of parents had a diagnosis of schizophrenia and were White (British).

The number of ward stays was originally non-significant (*t*(4495.8) = − 0.168, *p* = 0.8667) but became significant when excluding those who did not have a ward stay recorded (*t*(2552.7) = 1.988, *p* = 0.0458). The mean average number of ward stays between parents and non-parents was almost identical (1.89 for parents and 1.88 for non-parents); however, when excluding those without any ward stay recorded, the average ward stays of parents rises to 3.41 and non-parents to 3.71. IMD was not significant (*t*(4231.1) = − 0.650, *p* = 0.5155) and smoking status was initially significant (*X*^*2*^(3, *N* = 5173) = 10.73, *p* = 0.013) but became non-significant when those with missing data were excluded (*X*^*2*^(2, *N* = 2454) = 3.88, *p* = 0.144).

### Regression modelling

All ten participant-level variables (age, gender, ethnicity, marital status, accommodation, employment, diagnosis, ward stay, smoking and index of multiple deprivation) were included in the model. Table 2 presents the mutually adjusted odds ratio of being a parent for each variable in the model.

**Table 2:** Parenting status regression

Covariates	Odds ratio [Confidence intervals]	*p* value
Age	1.04 [1.04–1.05]	< 0.001**
Ward stay	1.03 [1.01–1.05]	0.015*
IMD group	0.95 [0.93–0.98]	< 0.001**
Marital status Compared to ‘single’		
Married	7.60 [6.13–9.41]	< 0.001**
Divorced	8.55 [6.54–11.18]	< 0.001**
Unknown	2.42 [2.01–2.93]	< 0.001**
Gender Compared to ‘male’		
Female	2.17 [1.88–2.50]	< 0.001**
Ethnicity Compared to ‘White – British’		
Asian or Asian British	1.34 [1.02–1.75]	0.036*
Black or Black British	1.42 [1.03–1.94]	0.030*
Mixed	1.42 [0.92–2.18]	0.114
White—other	1.13 [0.86–1.48]	0.384
Any other group	0.47 [0.25–0.86]	0.014*
Unknown	1.08 [0.86–1.36]	0.502
Accommodation Compared to ‘Supported Living’		
Owning	2.07 [1.44–2.98]	< 0.001**
Renting	2.32 [1.75–3.06]	< 0.001**
Temporary or prison	1.33 [0.95–1.86]	0.092
Unknown	1.47 [1.05–2.06]	0.026*
Employment Compared to ‘Unemployed’		
Employed	0.79 [0.53–1.20]	0.274
Retired	0.64 [0.41–0.99]	0.044*
Student	0.05 [0.01–0.24]	< 0.001**
Benefits	0.77 [0.52–1.15]	0.203
Unknown	0.69 [0.50–0.97]	0.030*
Diagnosis Compared to F20—schizophrenia		
F21	1.66 [0.65–4.23]	0.285
F22	1.35 [0.99–1.84]	0.057
F23	1.99 [1.50–2.64]	< 0.001**
F25	1.13 [0.90–1.42]	0.276
F28	1.88 [1.01–3.49]	0.047*
F29	1.45 [1.06–1.98]	0.021*
F31.2 and F31.5	1.51 [1.13–2.02]	0.005**
Psychosis indicated through cluster level	1.59 [1.29–1.97]	< 0.001**
Smoking Compared to ‘Non-smoker’		
Current smoker	1.84 [1.49–2.28]	< 0.001**
Ex-smoker	1.29 [0.94–1.79]	0.119
Unknown	1.15 [0.88–1.50]	0.309

A higher age and number of ward stays were both positively associated with parenthood. Participants living in less deprived neighbourhoods were slightly less likely to be a parent. Women in the sample were more than twice as likely to be parents as men in the sample. Patients who were married or divorced were more likely to have a child when compared with participants who were single. When compared to participants who were White—British, most other ethnicities had higher odds of being a parent. For accommodation, participants who were owning or renting were twice as likely as those in supported living to be a parent. Participants who were recorded as unemployed were more likely to be a parent than those who were students, retired or in employment, with students being the least likely group to have children. Participants with non-schizophrenia psychoses were also more likely to have children than participants with a diagnosis of schizophrenia. Current smokers were more likely to be parents than non-smokers.

The sensitivity analysis, i.e. parents with dependant children and non-parent participants, produced similar findings (see Supplementary table 3).

## Discussion

### Recording of patients’ children

The first aim of this study was to establish whether children are recorded in the correct place on patients’ clinical notes. Out of the 2006 parents that were identified, fewer than half of them had their children recorded in the appropriate structured field. Instead, the majority were identified by searching free-text notes and then reading through each note to confirm the presence or absence of a child. Even for the patients who had children entered in the appropriate field, the dates of birth of these children were often not recorded. Although dependants were more likely to be recorded than non-dependants, these findings nevertheless suggest that further work with staff is needed to meet the requirements for recording children accurately in this Trust [9].

### The proportion of those with a diagnosis of psychosis who are a parent

Over a third (38.8%) of the 5173 patients with psychosis were parents. More than half (53.9%) of female patients with psychosis were mothers and around a quarter (26.9%) of male patients with psychosis were fathers. The rate found in this study is very similar to the most recent international estimate conducted by a national survey in Australia, which found that 38.1% of the 1825 participants were parents, with 56.2% of women being mothers and 25.9% of men being fathers [10]. Due to increasingly shorter hospital stays and the usage of newer anti-psychotics within women with psychosis [15, 16], we expected that the result of this study would be higher than the estimate from a previous study conducted in the UK, which reported that 63% of women with psychosis were mothers [12]. The reasons for our lower estimate are currently unclear. We know that Howard et al. [12] made attempts to establish epidemiological representative cases, although the smaller sample size of 246 may have resulted in an overestimate of the true proportion. Recently, it has been shown that women with a psychotic diagnosis in a UK sample have a lower fertility rate than the general population [25]; however, this does not account for women who develop psychosis after becoming a mother. Indeed, another recent study in the US found similar rates of parenthood in a sample with serious mental illness compared to a sample without a mental health diagnosis [26].

### Factors associated with parenthood

An examination of the factors associated with parenthood found significant differences between parents and non-parents in age, gender, ethnicity, marital status, diagnosis, employment and accommodation. Older age, a higher number of ward stays, higher socioeconomic status, being female, renting or owning their home, having ever been married, not having schizophrenia, being unemployed, not being White British and being a current smoker were all important factors associated with parenthood. Many of these variables, such as being older or renting/owning a home, point towards a more settled lifestyle, possibly, giving individuals with psychosis more opportunities to meet a partner and have children.

The results from this model identified that women with psychosis were much more likely than men to be a parent. Other studies have also confirmed gender as an important variable in whether one has a child with a psychotic diagnosis [10, 12, 20]. More parents with psychosis have their first psychotic episode after becoming a parent rather than before [12, 18, 27]. Since psychosis has an earlier age of onset in men than in women, elements related to psychosis such as poverty and isolation [28] may provide men with fewer opportunities to have children. It might also be the case that the true incidence of parenthood within men with psychosis is under-reported. Since parenthood was recorded whenever there was a mention of children, and men with psychosis are less likely to have contact with their children [29], the parenting status of some men in this sample may have been missed.

Those with a diagnosis of acute psychotic disorder (F23) were much more likely than those diagnosed with schizophrenia (F20) to have children and those with bipolar disorder with psychotic symptoms (F31.2 or F31.5) were also more likely. Schrank et al. [20] also found that those with a diagnosis of schizophrenia were the least likely to have children when compared to other psychotic diagnoses. To be diagnosed with schizophrenia, both positive and negative symptoms must be present. Individuals with schizophrenia are also more likely than those with another psychotic disorder to experience cognitive symptoms and for these symptoms to be chronic rather than episodic [30]. The differences between these diagnoses in this study might reflect the fact that a higher symptom severity can lead to fewer opportunities to meet a partner and have children.

The missing indicator method was used to address the missing data in the regression analysis. It is not possible to prove whether data are missing at random (MAR) or missing not at random (MNAR) without follow-up of participants, which due to the design of this study is not possible. However, there is strong reason to hypothesise that the data were MNAR, and thus the missing indicator method was appropriate, due to the nature of administrative data. For example, there were high levels of missing data for smoking status. It is well known that smoking status is more likely to be missing for non-smokers than smokers in health records [31]. This study had 23% of participants recorded as smokers. In the UK, around 37% of those with a mental health condition are smokers [32], meaning the large amount of missing data in this variable (47%) is likely to be mostly attributable to non-smokers rather than smokers. Therefore, it seems likely that non-smokers were less likely to have their smoking status recorded and that the missingness within the smoking status variable was attributable to smoking status itself.

### Strengths and limitations

A strength of this study is that it updated the estimate of the proportion of patients with psychosis who are parents in the UK, based on data from an NHS Trust sample. Extensive work was done to identify children by reading the clinical notes of patients. However, due to the time it took to identify parent status in this study’s cohort, it was not possible to perform an analysis whereby this cohort of parents with a psychotic diagnosis was matched with another cohort of parents (e.g. parents with a diagnosis of depression), to examine whether the sociodemographic factors identified are specific to psychosis.

Using administrative data meant the study was limited to the information completed by healthcare professionals. This resulted in a large amount of missing data, especially in relation to employment, accommodation and smoking status. Including more factors about the child, such as contact with child and family services, accessing child mental health services, and education level, may have informed this analysis, but these variables are not available when using the CRIS dataset.

Due to the data likely being MNAR, and the outcome in this study (parenthood) being common, the estimates obtained may be biased, and therefore, should be interpreted with caution with focus on the directionality rather than specific estimates obtained. The aim of this study was to identify characteristics amongst patients with psychosis that are associated with parenthood, and therefore, another limitation is that no conclusions can be drawn about the temporal relationships between each independent variable and parenthood.

### Implications for practice and research

Although the Think Family initiative [9] recommends the recording of children on patients’ clinical notes, the current findings suggest that this may still not be happening systematically and comprehensively. Children of parents with psychotic disorders are at risk of emotional and behavioural difficulties during childhood [33] and psychopathology later in life [34]. Interventions currently exist to provide support to these children by explaining their parent’s illness [35], linking the family with a caseworker [36], and providing treatment for children’s own mental health difficulties [37]. However, before receiving support, these children must be accurately identified, and that information should also be shared between adult mental health services and other agencies [38]. We can see from global examples that it is possible to increase the identification of these children. For example, after changes to legislation and the introduction of the ‘Assessment Form’ in Norway, rates of identification of patients’ parenting status increased [6].

This study has established that within the UK, over a third of patients with a psychotic diagnosis will also be a parent. Healthcare professionals working with these patients would likely benefit from training in providing family-focussed care which may include recognising the centrality of patients’ parenting role, providing age-appropriate information to children, creating a family-friendly environment during inpatient visits and referring families to additional supports [39]. This study also highlighted that some patients with psychosis are more likely to be parents than others, including those who are female and of an older age. This information will help healthcare professionals in targeting interventions and support to certain client groups.

CRIS provides large datasets representing an anonymised form of a patient’s clinical notes. Future research on parents with psychosis and their children could benefit from using additional CRIS datasets from more than one Trust and enabling natural language processing to improve the identification of children in patients’ notes. The CRIS dataset could also be linked with other datasets to obtain more details on the parents and their children; for example, with the Hospital Episode Statistics (HES) database [40], which would allow all children born to the parent to be identified.

## Supplementary Information

Below is the link to the electronic supplementary material.

Supplementary file1 (DOCX 20 KB)

## Data Availability

Data are not publicly available and can only be accessed using CRIS, which provides access to anonymised data from patients’ clinical records. For more information, please contact the CRIS Administrator at CRIS.Admin@oxfordhealth.nhs.uk.

## Code availability

The R code used in this analysis is available here https://github.com/JessRadley/CRIS-parents-with-psychosis.
