# Supplementary material for: Sociodemographic characteristics associated with parenthood amongst patients with a psychotic diagnosis: a cross-sectional study using patient clinical records
Source: Soc Psychiatry Psychiatr Epidemiol. 2022 Apr 21;57(9):1897–906. doi: 10.1007/s00127-022-02279-x (PMC9375763; doi:10.1007/s00127-022-02279-x)
Supplement: Supplementary file 1 — Supplementary file1 (DOCX 20 KB) [file 127_2022_2279_MOESM1_ESM.docx]

# Supplementary material

**Article Title:** Sociodemographic characteristics associated with parenthood amongst patients with a diagnosis of psychosis: A cross-sectional study using patient clinical records

**Journal Name:** Social Psychiatry and Psychiatric Epidemiology

**Author names:** Jessica Radley, Jane Barlow, Louise C. Johns

**Affiliation and e-mail address of corresponding author:** Department of Psychiatry, University of Oxford, Warneford Hospital, Warneford Lane, Oxford, OX3 7JX, UK, [jessica.radley@psych.ox.ac.uk](mailto:jessica.radley@psych.ox.ac.uk)

Supplementary table 1 - Number of children and ages of children per patient

|  |  | **Total = 2006** |
| --- | --- | --- |
| Number of children | One | 914 (45.56%) |
|  | Two | 684 (34.10%) |
|  | Three | 264 (13.16%) |
|  | Four | 87 (4.34%) |
|  | Five | 36 (1.79%) |
|  | Six | 13 (0.65%) |
|  | Seven-Twelve | 8 (0.40%) |
| Under/Over 18 | Any children under 18 | 740 (36.89%) |
|  | All children 18 and over | 1118 (55.73%) |
|  | Unknown | 148 (7.38%) |

Supplementary table 2 - Child demographics

|  |  | **Total = 3745** |
| --- | --- | --- |
| Gender | Male | 1689 (45.10%) |
|  | Female | 1831 (48.89%) |
|  | Transgender | 1 (0.03%) |
|  | Unknown | 224 (5.98%) |
| Over/Under 18 | 18 and over | 2236 (59.71%) |
|  | Under 18 | 1189 (31.75%) |
|  | Unknown | 320 (8.54%) |
| Ages | 0-2 | 86 (2.30%) |
|  | 3-6 | 209 (5.58%) |
|  | 7-11 | 310 (8.28%) |
|  | 12-15 | 256 (6.84%) |
|  | 16-17 | 123 (3.28%) |
|  | 18 and over | 690 (18.42%) |
|  | Exact age unknown | 2071 (55.30%) |

Supplementary table 3 - Parenting status (dependants) regression

| **Covariates** | **Odds ratio [Confidence intervals]** | **p-value** |
| --- | --- | --- |
| Age | 0.99 [0.98-1.00] | 0.004** |
| Ward stay | 1.02 [1.00-1.05] | 0.088 |
| IMD group | 0.93 [0.90-0.97] | <0.001** |
| Marital Status  *compared to ‘single’* | | |
| Married | 10.35 [7.89-13.58] | <0.001** |
| Divorced | 6.98 [4.70-10.35] | <0.001** |
| Unknown | 2.16 [1.70-2.75] | <0.001** |
| Gender  *compared to ‘male’* | | |
| Female | 1.92 [1.59-2.32] | <0.001** |
| Ethnicity  *Compared to ‘White – British’* | | |
| Asian or Asian British | 1.44 [1.05-1.96] | 0.022* |
| Black or Black British | 1.32 [0.88-1.99] | 0.181 |
| Mixed | 1.43 [0.88-2.32] | 0.150 |
| White – Other | 1.26 [0.90-1.77] | 0.184 |
| Any other group | 0.62 [0.29-1.30] | 0.206 |
| Unknown | 1.09 [0.79-1.49] | 0.602 |
| Accommodation  *Compared to ‘Supported Living’* | | |
| Owning | 1.98 [1.12-3.49] | 0.018* |
| Renting | 2.14 [1.40-3.28] | <0.001** |
| Temporary or prison | 0.90 [0.55-1.47] | 0.677 |
| Unknown | 1.28 [0.76-2.13] | 0.353 |
| Employment  *Compared to ‘Unemployed’* | | |
| Employed | 0.84 [0.54-1.32] | 0.455 |
| Retired | 0.04 [0.01-0.17] | <0.001** |
| Student | 0.02 [0.00-0.17] | <0.001** |
| Benefits | 0.65 [0.41-1.03] | 0.066 |
| Unknown | 0.61 [0.42-0.88] | 0.008** |
| Diagnosis  *Compared to F20 – schizophrenia* | | |
| F21 | 1.73 [0.45-6.68] | 0.428 |
| F22 | 1.67 [1.07-2.62] | 0.024* |
| F23 | 2.29 [1.62-3.23] | <0.001** |
| F25 | 1.16 [0.84-1.59] | 0.375 |
| F28 | 1.61 [0.78-3.34] | 0.197 |
| F29 | 1.80 [1.26-2.58] | 0.001** |
| F31.2&F31.5 | 1.72 [1.18-2.50] | 0.004** |
| Psychosis indicated through cluster level | 1.67 [1.27-2.20] | <0.001** |
| Smoking  *Compared to ‘Non-smoker’* | | |
| Current Smoker | 1.59 [1.21-2.10] | 0.001** |
| Ex-smoker | 1.51 [1.00-2.28] | 0.051 |
| Unknown | 1.05 [0.72-1.52] | 0.819 |
